# Indigenous Land-Based Approaches to Well-Being: The *Amisk* (Beaver) Harvesting Program in Subarctic Ontario, Canada

**DOI:** 10.3390/ijerph19127335

**Published:** 2022-06-15

**Authors:** Fatima Ahmed, Eric N. Liberda, Andrew Solomon, Roger Davey, Bernard Sutherland, Leonard J. S. Tsuji

**Affiliations:** 1 Department of Physical and Environmental Sciences, University of Toronto, Toronto, ON M1C 1A4, Canada; 2 School of Occupation and Public Health, Faculty of Community Services, Ryerson University, Toronto, ON M5B 2K3, Canada; eric.liberda@ryerson.ca (E.N.L.); leonard.tsuji@utoronto.ca (L.J.S.T.); 3 Fort Albany First Nation, Fort Albany, ON P0L 1H0, Canada; andrewsolomon83@gmail.com (A.S.); roger.daveyfafn@gmail.com (R.D.); 4 Peetabeck Academy, Mundo Peetabeck Education Authority, Fort Albany, ON P0L 1H0, Canada; fatima1.ahmed@ryerson.ca

**Keywords:** Indigenous, Canada, well-being, on-the-land activities, traditional activities, multiple perspectives, cortisol, photovoice, environment

## Abstract

The act of decolonizing knowledge systems involves recovering and renewing traditional, non-commodified cultural patterns, such as the sustenance of intergenerational relationships and traditional practices. A decline in beaver harvesting, which was once an integral part of the *Omushkego* Cree culture, has resulted in an overabundance of beavers and dams, which has negatively affected communities by increasing the local flooding events and impacting the water quality. The aim of the *Amisk* (beaver) program was to reconnect the Elders and youth to revitalize traditional on-the-land activities and, in the present case, beaver harvesting and associated activities within the community. The program and evaluation were built using a two-eyed seeing (*Etuaptmumk*) and community-based participatory research approach. Salivary cortisol, a biomedical measure of stress, was collected before and after participation in the program. Photovoice, along with semi-directed interviews, were employed to identify the key elements of well-being from a First Nations’ perspective. For the beaver harvesting activities, the changes observed in the cortisol concentrations were not statistically significant (*p* = 0.094). However, the act of beaver dam removal was associated with a statistically significant increase in the post-participation cortisol concentration (*p* = 0.021). It was noteworthy that increased stress during the removal of the beaver dams–as indicated by the elevated post-activity cortisol levels–were not reflected in a decrease in the qualitative measures (semi-directed interviews and photovoice) of well-being from an Indigenous perspective. In fact, there was a noted increase in the subjective well-being of the participants, which highlights the importance of multiple perspectives when assessing well-being, especially in Indigenous peoples. However, the cortisol findings of the present pilot project need to be interpreted with caution, due to the limited sample sizes.

## 1. Introduction

Indigenous knowledge systems and teachings have been integral to preserving Indigenous cultures and traditions. Meanwhile, colonization has negatively impacted Indigenous cultures, resulting in a decrease in traditional activities [1]. To rebuild the traditional knowledge systems and regain what was lost due to colonial assimilative policies (e.g., residential schools) [2], the younger generations need to be reconnected to their Elders and the land [3,4]. Increased participation in traditional practices and partaking in a traditional diet has been shown to not only provide physical benefits but also create positive impacts on the overall well-being of the individuals and communities [5]. 

In the western James Bay region of northern Ontario, Canada, the *Omushkego* Cree have historically utilized the major water bodies, such as James Bay, the Albany River, Moose River, and Attawapiskat River. These waterways have served as a means for transportation and have a deep-rooted cultural and historical significance for the community [6,7]. Due to the James Bay region being located in subarctic Canada, foods, such as fruits and vegetables, are more expensive to obtain as they must be flown into the communities, and crop production under ambient conditions is seasonally limited [8]. This has affected the availability of safe and nutritious food. At the grocery store, readily available, prepackaged foods have been found to be higher in refined sugars and saturated fats [9]. The frequent consumption of these foods has led to higher rates of diabetes and cardiovascular disease within the communities [10,11,12]. Alternatively, traditional food, obtained through hunting, trapping, fishing, and gathering, is another source of sustenance. Historically, trapping was an integral part of the James Bay region, including the harvesting of beaver (*Castor canadensis*), which provided both food and pelts, being economically beneficial on several levels to the First Nation communities [4,13]. It should be noted that traditional meats have been shown to be rich in essential vitamins and nutrients, which are relatively limited in store-bought food, as mentioned previously [9]. For this reason, a decrease in the consumption of traditional foods has partly contributed to the increased prevalence of chronic diseases within this region [10,11,12].

At present, the trapping of beaver is infrequent in the western James Bay area, leading to an increased population of beavers in this region [14]. This has been a contributing factor to the environmental concerns of local flooding. Over the last two decades, annual flooding on a regional basis has been raised as a major concern and continues to be a reoccurring problem [14,15,16]. Overall, environmental change and a decline in subsistence practices, which were a source of beaver management, contribute to the flooding [14]. Increased temperatures and excessive rainfall in the spring have caused quicker snow melts, resulting in larger amounts of water flowing down the river and causing flooding [14,15,16]. These flooding events have also been attributed to the overpopulation of beavers and to beaver dams [14].

Beaver dams are interwoven structures built from rocks, logs, grass, and mud, to stop the water along streams [6,17]. The resulting pond provides a habitat for the beaver and allows it to transport logs and branches in the summer [17]. The lodge is the main shelter for the beaver family unit, also known as a colony, and protects it from predators and weather conditions [17]. The food caches for these lodges contain submerged branches, which are stored in the fall and consumed in the winter [17]. While harvesting, the beavers create paths, such as trails while dragging tree limbs, and canals, which are dredged channels filled with water [17]. These activities can cause prolonged flooding [17,18]. The contamination of the water systems from beaver feces also creates an increased risk of diseases, such as cholera, *Escherichia coli (E. Coli)*, leptospirosis, and respiratory infections [19,20]. In the Kashechewan First Nation (FN), the beaver colonies blocked the drainage systems leading out of the sewage lagoon, causing it to back up into the creek and become contaminated with *E. Coli*, which can be deadly to human beings dependent on the strain [14,21]. South of Kashechewan FN, in Fort Albany FN, flooding during the spring melt, and yearly evacuations are not uncommon [14,16,19]. These evacuations cause significant damage to the housing within the community and, as seen in the 2008 flood, restrict access to the local hospital, school, and airport [15]. These issues not only create economic challenges for the communities and government, but negatively impact the environment, wildlife, and cause a great deal of psychological and physical stress on the residents of this region [14]. Unfortunately, the frequency and risks associated with these evacuations are increasing, due to the impacts of climate change, changes in the weather patterns, and the overpopulation of beavers [14,19,22]. 

Since time immemorial, the populations of beavers have been maintained through Indigenous harvesting practices [23,24], and, more recently (i.e., the 1600s), the sale of the pelts to the Hudson’s Bay Company [13,25]. However, beaver harvesting, once an integral part of the *Omushkego* Cree culture, has declined significantly, due to the changes stemming from colonization, environmental change, and technological advances [11,24,26]. This decline has caused an increase in the population of beavers, which has led to more frequently flooding events, risks to water quality and, overall, negative impacts on communities and the environment [17,23]. Addressing these environmental issues requires cooperation between the different levels of Canadian government and Indigenous knowledge holders to effectively deal with these complex environmental problems [27,28,29]. To that end, there is an opportunity for the revitalization of subsistence activities, such as beaver harvesting, to address in part the local flooding issue, while also providing other health and well-being benefits (e.g., social, cultural) for the harvesters.

Well-being can be viewed both objectively through measures of health, and also subjectively, through measures such as an individual’s perspective and experience of their life [30]. In Canada, the Community Well-Being Index was developed using four parameters (i.e., education, labor force activity, income, and housing) to derive a community well-being score [12,31]. The scores were used to compare well-being across the Indigenous and non-Indigenous communities. However, there was no consultation with the Indigenous people, nor were culturally relevant variables included [12]. There was increasing awareness that health and well-being are more than a biomedical concept, being also determined by the social circumstances and cultural contexts [32]. This approach to health and well-being is relatively new in western scientific research, however, has always been a part of Indigenous teachings. As stated in many studies, Indigenous health and well-being cannot be measured in the absence of Indigenous knowledge and relationship with the land [14,26,30,32,33,34,35]. Therefore, it is integral to include the Indigenous knowledge holders through methods such as community-based participatory research, which allows for the inclusion of community members at all of the stages. 

Another method, which can measure the impacts of colonization and the subsequent health and well-being disparities faced by the Indigenous communities, is the measurement of stress through the determination of cortisol levels [1,36,37,38]. Cortisol homeostasis is impacted through either external or internal stressors [1,39,40]. Further, chronic stress over a long period can negatively impact an individual’s health and well-being, resulting in a variety of negative outcomes. Many of the Indigenous communities experience stress related to socioeconomic factors, disease, intergenerational trauma, the environment, and other health and social inequities [1,36,37,38,39,41,42,43,44]. An unequal exposure to stressful social and environmental factors shapes how the brain processes stimuli and has been shown to underlie health disparities [42]. Henley, Jahedmotlagh, Thomson, Hill, Darnell, Jacobs, Johnson, Williams, Williams, Van Uum, Bend and Koren [37] report that approximately one-third of the Indigenous peoples in Canada aged 15 and older have been diagnosed with a chronic health problem. Since increased levels of cortisol have been implicated in the development of many health conditions, quantifying cortisol levels offers an insight into the overall health and well-being [44]. Chronic stress has also played a major role in the development of somatic and mental disorders [1,42].

Stress has been suggested as a key determinant of health and well-being in the Indigenous communities [37]; however, a knowledge gap remains in exploring effective programs/interventions to reduce the levels of stress within these populations [42]. Levels of stress have also been shown to be contributing factors to the high rates of health and well-being disparities in communities [36,41,42]. Therefore, there is a need for multi-perspective approaches to the health programs/interventions aimed at addressing the health and well-being inequities in the Indigenous communities. Increasing evidence has shown the importance of using approaches such as two-eyed seeing (*Etuaptmumk*), which uses Indigenous and mainstream western knowledge systems as complementary constructs to address the needs of the Indigenous communities [45]. This study was developed using these methods to address significant gaps in the literature, and, more importantly, to build on community wishes, to explore the perceptions and experiences of the participants in a beaver-harvesting-revitalization program in Fort Albany FN, northern Ontario, Canada. Thus, the objectives of this study were: to revitalize on-the-land beaver harvesting activities and to reconnect the youth with the land; protect the community from local flooding; enhance local water quality; and contribute to the well-being of the participants and their community.

## 2. Materials and Methods

### 2.1. Study Design and Participants

We utilized the two-eyed seeing (*Etuaptmumk*) and community-based participatory (CBPR) approaches. The two-eyed seeing approach stresses the importance of viewing through both the mainstream western and Indigenous worldviews for the greater good of everyone [45,46,47]. Community-based participatory research is “a practical approach to problem solving through planning, action and reflection” addressing social problems [34,48] and recognizing communities as a unit of identity, to build on their strengths and resources [48]. Using the complementary approaches, such as the two-eyed seeing approach and CBPR, to better understand health and well-being allowed for the inclusion of the diverse perspectives and experiences of community members.

This project was developed in partnership with the Fort Albany First Nation leadership (A.S.), and the community-based coordinators (R.D., B.S.). These individuals, along with other Fort Albany FN Band Council members, provided direction at each stage including the selection of Elders and other on-the-land experts to lead the project, as well as the youth participants; and the general planning of the activities, such as, when, where, and how the harvest would take place; and what information would be shared with the non-Indigenous research team members. The participants were ≥18 years of age.

#### 2.1.1. Beaver Harvesting

The beaver harvesting occurred in the winter of 2018, with activities ranging from 3 days to 1 week. The salivary cortisol samples were taken the morning prior to commencing the activity and the morning after completion of the activity. The Elders and on-the-land experts led the youth participants to trap lines around the community, including the Albany River and its tributaries, where the youth participants were taught methods of beaver trapping. A combination of conibear and snare traps were used during the trapping, with instructions on safety measures being provided by the Elders and on-the-land experts throughout. These individuals also determined the type of traps used, the location, the lure, and the preparation and setting of the trap. The youth participants were also taught methods of skinning and smoking beavers; however, these activities were not formally tracked for this study. All of the beavers obtained through this project were shared by the participants and the Band Council with the community.

#### 2.1.2. Beaver Dam Removal

The beaver dam removal occurred in the fall of 2018, with activities ranging from 3 days to 1 week. The salivary cortisol samples were taken the morning prior to commencing the activity and the morning after completion of the activity. The Elders, on-the-land experts, and the Band Council advised on which of the beaver dams needed to be removed around the community to restore the waterways to a more natural state to alleviate community flooding. To ensure that any remaining beavers, who could potentially rebuild the dams after dam removal, were not a continuing nuisance, the beavers were harvested utilizing conibear and snare traps. Although these methods were similar methods to the beaver harvesting project, there were some variations to account for the lack of snow and ice cover. The youth participants were taught by the Elders and on-the-land experts how to remove the beaver dams safely, and the environmental context was explained in detail. All of the beavers obtained through this project were shared by the participants and the Band Council with the community.

#### 2.1.3. Consent

The process for obtaining consent was completed in a manner which was inclusive and transparent. The participants were gathered in a neutral space to discuss the study. These discussions were in English with translators if their preferred language was Cree. The participants were advised that their involvement was entirely voluntary, and they were able to withdraw at any time. The participants were also able to opt-out of any part of the research if they chose (i.e., not providing saliva samples). The OCAP^®^ (Ownership, Control, Access, and Possession) principals established how the data and information were collected, protected, used, or shared. The participants were assigned a random number which was only identifiable by the principal investigator and the community coordinator. Informed consent was given by all participants in the study. Ethics approval was obtained through the University of Toronto and Ryerson University ethics committees.

### 2.2. Data Collection

#### 2.2.1. Salivary Cortisol

The saliva samples were collected from the participants using Salivette^®^ cotton swab tubes (Sarstedt, Numbrecht, Germany) in the mornings upon waking, prior to consuming any food, drink, or smoking. These sample tubes were provided pre-labelled to each participant. These samples were taken prior to and after participation in each activity. Once samples were collected, they were stored in a −20 °C freezer by the community coordinator. They were then transported in a cooler to the University of Toronto laboratory, where they were stored at −20 °C until processing.

#### 2.2.2. Semi-Directed Interviews

The semi-directed interviews allowed the participants to share their perspectives on individual well-being and factors which affect it. The list of questions included: 1. What is well-being? 2. What is important for your well-being? 3. Can well-being be measured? 4. Are there barriers to well-being? 5. Are there opportunities to achieving well-being? 6. Does being on the land contribute to well-being? 7. Are there any other comments that you would like to make that were not covered by the previous questions? These questions were formulated with and approved by our First Nation Advisory Committee. The interviews were conducted and recorded in a neutral setting and, if required, with an English/Cree translator of their choice. The interviews were all recorded on data recorders, and transcribed verbatim. The interviews are kept at the University of Toronto on encrypted computers, which are only accessible to the authors.

#### 2.2.3. Photovoice

For a second qualitative measure, photovoice was selected. Photovoice (or photo novella) was first reported by Wang and Burris [49,50], with the idea that a foundation of images and words enable participants to connect with policymakers, by allowing them to articulate their concerns through a visualization of their lived experience. Wang and Burris [49] stated that the images allow for statements to be made, which cannot be expressed through just words alone. Photovoice allows individuals to be active participants rather than the objects of study [49], which empowers vulnerable populations [34], and allows for the co-creation of knowledge through dialogue [51]. Additionally, photovoice engages individuals through questions that reflect on their community issues, historic, and social situations [52]. Lastly, this type of documentary photography is based on the premise that individuals become empowered to reflect on and instill change in their communities through visuals [34,53]. Thus, photovoice is a successful and effective method to empower marginalized communities to address the inequities they face [34].

These methods were selected for this study as the methods were easy for the participants to learn and empowering; the participants were given the opportunity to present their perspectives and experiences with regards to well-being. Each participant was provided with a GoPro Hero5 (GoPro, Inc., San Mateo, California, United States of America (USA)) and instructed to take photos of anything that was associated with well-being or wellness from their perspective. One camera was distributed to each individual; the participants were given a brief demonstration on camera operation and an opportunity to practice. At the end of each month, the photos were all downloaded onto a hard drive and stored securely, physically, and electronically, at the University of Toronto. Once these were obtained, the participants provided a narration of their photos in a neutral setting. In the same way as the semi-directed interviews, the participants were able to conduct the interview in English or Cree, with a translator of their choice. The length of the discussions varied depending on the number of photos; the length and depth of discussion varied with each individual. The participants who did not collect any photos were still invited to take part in the semi-directed interviews.

### 2.3. Data Analysis

#### 2.3.1. Salivary Cortisol

The cortisol samples were collectively analyzed by In-Common Laboratories (ICL). ICL (Toronto, ON, Canada) which is a medical testing laboratory licensed by the Ontario Ministry of Health and Long-Term Care. It is also an accredited by the Institute of Quality Management in Healthcare and holds the 15189 PlusTM certificate. The lab completed an ElectroChemiLuminescence Immunoassay (ECLIA) analysis using Cobas e 411 Analyzer (Roche Diagnostics GmbH, Mannheim, Germany). This method provides a high level of accuracy and a wider detection range than previously employed methods [54]. The Detect Limits (DL) for the salivary cortisol concentrations were 1.5 nmol/L. The inter-assay coefficients of variation at high and low concentration were 12.53% and 8.98% respectively. An intra-assay coefficient of variation was not available.

The data were analyzed using the Statistical Package for Social Sciences (SPSS) 13.0 (SPSS, Inc., Chicago, IL, USA). The cortisol measurements were assessed for normality using the Shapiro–Wilks’s test and then log transformed. Pre- and post- concentrations were compared using a Paired *t*-test (two-tailed) to examine whether the differences were statistically significant. Following Jung, et al. [55], we input samples below the detection at their DL value of 1.5 nmol/L. A sensitivity analysis to examine the effect of utilizing half of the DL did not alter the significant findings. A *p* value < 0.05 was considered significant.

#### 2.3.2. Semi-Directed Interviews and Photovoice

The analysis of the qualitative data was based on the grounded theory and the constant comparative method [56,57]. The grounded theory is a systematic method which involves the construction of a theory through an analysis of data [57]. A constant comparative method, derived from the grounded theory [56], involves “multiple stages of collecting, refining and categorizing the data” [57]. The four stages of this method include “comparing incidents applicable to each category, integrating categories, delimiting the theory and writing the theory” [57]. This method is effective as it allows for the use of raw data and, through constant comparisons, allows for the generation of a new theory.

The qualitative data from the Interviews and photovoice narrations were transcribed manually using NVivo 11 (QSR International Pty, Melbourne, Australia) software analyzer. The thematic analysis of the data began through immersion in the data. This including reading transcripts multiple times after transcriptions, followed by an analysis of individual transcripts to identify phrases, sentences, or paragraphs of importance to the participants. These findings were subsequently coded through NVivo 11 (QSR International Pty, Melbourne, Australia). After this stage, another level of coding occurred to determine the relationships between the codes to allow for the development of categories. The literature was revised, and the categories and overall coding were then revised and organized into themes by activity (beaver trapping and beaver dams). English translations were used for the interviews that were completed in Cree.

## 3. Results

A total of 14 participants were involved in this study, with one additional interview with an employee at the water treatment plant who is also a member of this Indigenous community. Of the total participants, three of participants were Elders, four were on-the-land experts and seven of the participants were youth. The participants in this study were males, with ages ranging from 18 to 80. The following sections draw on the two-eyed seeing approach, highlighting two perspectives, the biomedical and the First Nations.

### 3.1. Salivary Cortisol

**Figure A1:**
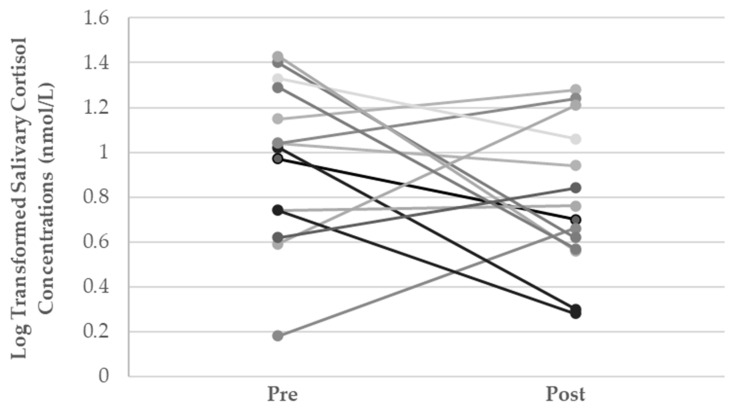
Plot showing changes in salivary cortisol concentrations pre- and post-participation in beaver harvesting activities for all participants.

Results of the within-group comparisons of salivary cortisol are displayed in Figure A1 (please see Appendix A) and Table 1. As stated previously, not all of the salivary cortisol samples for each participant were able to be quantified. A total of 39 samples were collected for this project. The post activity samples that were not submitted by the participant were removed from the analysis.

#### 3.1.1. Beaver Harvesting Activities

**Figure A2:**
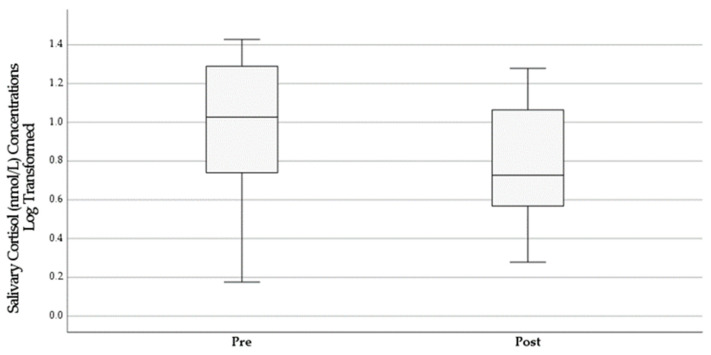
Box plot showing changes in the mean salivary cortisol concentrations for beaver harvesting activities.

The mean difference between the pre- and post-participation cortisol was 0.18 ± 0.48 nmol/L, which was not statistically significant (*t* (13) = 1.39, *p* = 0.094, two-tailed) (Figure A2).

#### 3.1.2. Beaver Dam Removal

**Figure A3:**
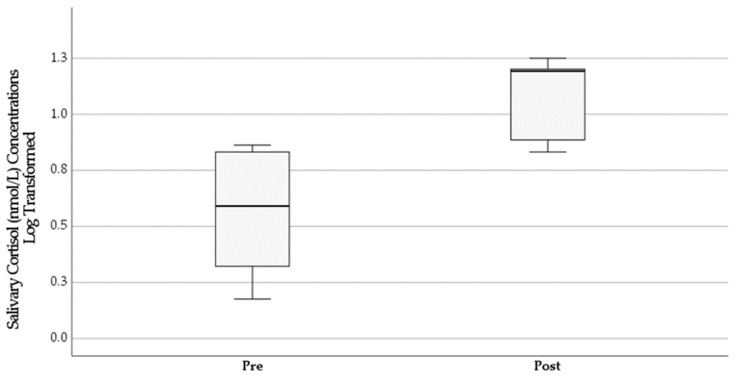
Box plot showing changes in the mean salivary cortisol concentrations for beaver dam breaking.

**Figure A4:**
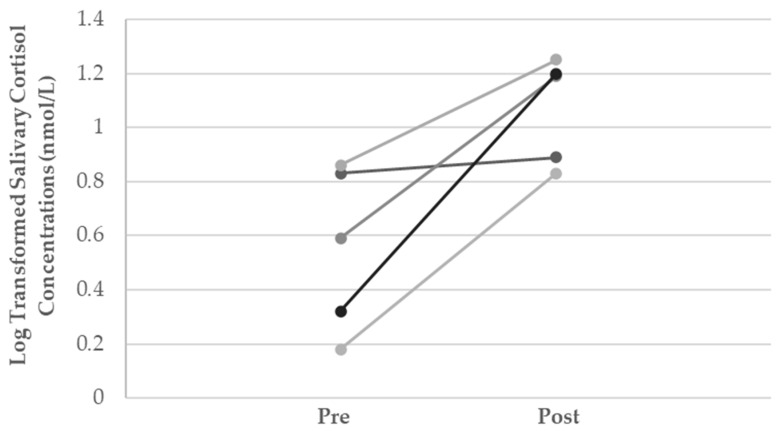
Plot showing changes in salivary cortisol concentrations pre- and post-participation in beaver dam breaking for all participants.

The mean difference between the pre- and post-participation cortisol were −0.52 ± 0.31 nmol/L, which was statistically significant (*t* (4) = −3.70, *p* = 0.021, two-tailed) (Figure A3). The negative effect means that the samples taken post-participation in the activity had higher concentrations than the samples taken before participating in the activity (Figure A4).

### 3.2. Semi-Directed Interviews and Photovoice

A total of 19 interviews, including the semi-directed interviews and the photovoice narrations, were conducted. One additional interview was completed with an employee at the water treatment plant for his observations. The following sections include quotes from the participants, and the images presented were censored to maintain anonymity. The themes presented and supported by selected verbatim quotes captured the views, feelings, and opinions of the participants. The images presented were censored, and any personal identifiable information was removed to maintain anonymity. The additional quotations, which were relevant but not featured in text, can be found in Appendix A (Table A1). The narratives provided by the participants helped to expand our understandings of well-being and how it not only contributes to the individual and social constructs within the communities, but also to the revitalization of traditional harvesting practices which contribute to the overall conservation of the surrounding environmental systems.

#### 3.2.1. Beaver Harvesting

The major themes identified were: (1) Knowledge; (2) Identity; (3) Healing; and (4) Land.

To understand the environmental context of the programs, an interview was conducted with an employee at the water treatment plant to discuss his personal experiences,

*“I’ve been doing so for about 20 years, and I have seen a variety of changes in our water regarding wildlife here and around our lakes … But mostly the beavers affected our water quality. Both in the raw and treated, so during the winter months the beavers would build dams around the creek, running from the lake to the river and that creek would run water from the lake to the river and so when the beavers build their dams, it constricts the flow from the lake to the river therefore it would cause a chain reaction… The lake would be constricted with flow and then we would get excess oxygen levels, and these oxygen levels would therefore produce iron manganese, and the iron-manganese would then get into our water treatment and then we treat it, but they are in suspended form until we treat it with chlorine. Then it comes out, then it precipitates out of, into solution, right? And then turns the treated water yellow…then I get a lot of complaints in town saying what’s going on… I try to explain to them, that the beavers are wreaking havoc on our lake”*

(Participant 37, Water treatment plant employee).

Local flooding was an initial community concern; however, through discussions, it was evident that the quality of the water was also being impacted. These discussions with the participants who reiterated their concern for the issues relating to the environment, local flooding, water quality and the overall well-being of individuals, emphasized the importance of this program, and other culturally relevant programs for the Indigenous communities.

**Knowledge**

Sharing Indigenous Knowledge

An important aspect of the beaver harvesting is the sharing of Indigenous knowledge, which has been passed down between generations through stories or through oral history and through observational learning. The sharing of knowledge was described as an important aspect of being on the land by a participant who stated that well-being was *“growing up healthier, and then sharing it to the younger people”* (Participant 33, Expert). These sentiments were shared by another participant, who stated, *“I was enjoying teaching those students”* (Participant 1, Expert). Statements such as these highlight the idea that well-being for individuals is not just limited to themselves, but to the well-being of the community as well. For many of the Elders and on-the-land experts, they were pleased to see youth partaking in cultural activities and expressed the desire to see more on-the-land programs in the community:

*“I think there should be more programs like the one that they did this winter for the young people, especially in the summertime like teaching them how to set nets and catch fish and how to prepare …then if you’re going to take them out on the land, if you take 10 people out there maybe 2 will become full-time so they can pass on the knowledge, that’s what we need, it’s important to pass it on those traditional skills”*

(Participant 3, Elder).

When speaking about their experience, many of the youth participants reflected on the knowledge they gained, *“It was awesome man, actually got to learn a few things”* (Participant 8, Youth). Similarly, another stated, *“I felt good about learning different things that I didn’t know, that I should know maybe for one day”* (Participant 16, Youth). As the Elders and on-the-land experts led the direction of the programs, the youth participants obtained an experience rich with a diverse set of knowledge and skills. One of the Elders discussed a misconception amongst some of the youth regarding the Elders and traditional knowledge:

*“I think there’s sometimes misunderstanding…sometimes people look at the Elders just because they never went to school, they don’t have the knowledge…and that’s wrong, eh? Because their language is in the environment, so everything they say is about the land”*

(Participant 3, Elder).

**Figure 1:**
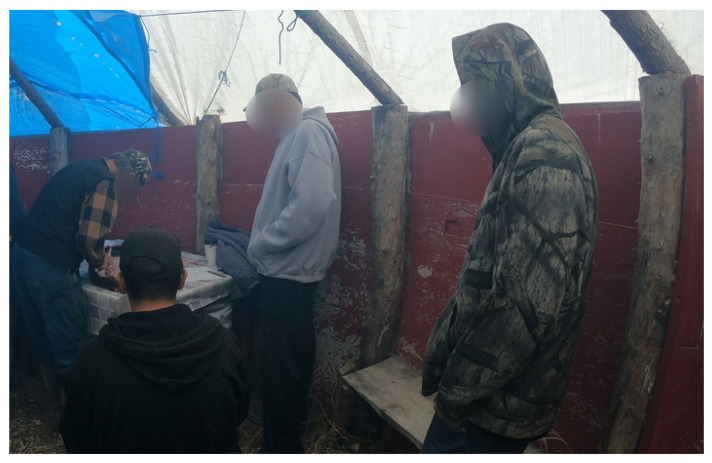
Youth and experts observing the skinning and smoking process for the beaver by an Elder.

Many of the youth participants expressed different sentiments regarding their relationships with the Elders (Figure 1):

*“It was really good…the much older they are, the more experience they can teach you everything and they don’t teach you by telling, they show you…and that’s the good thing about being out there with older people… if you go out there with an Elder like (omitted), which I haven’t really gone with him throughout the trapping …I hear that it’s real good learning from an Elder, they help a lot, and they can tell you what to do”*

(Participant 21, Youth).

Not only was it identified as an integral learning experience, but many were also proud of what they had learned and expressed their desire to share it with others,

*“Maybe one day if I go hunting with my Mooshum, maybe a cousin, or maybe a friend or a relative or somebody I know, that maybe wants to go out… I can show what I learned”*

(Participant 16, Youth).

Similarly, another youth participant shared, “*The teachings are passed down to the young ones and then as they grow, they can pass it down to their young ones”* (Participant 21, Youth).

As many of these statements centered around their ideas of well-being and the opportunities to achieve it, we can deduce that obtaining knowledge and sharing it with others was an integral part of this cultural experience for the participants.

Familial and Social Supports

**Figure 2:**
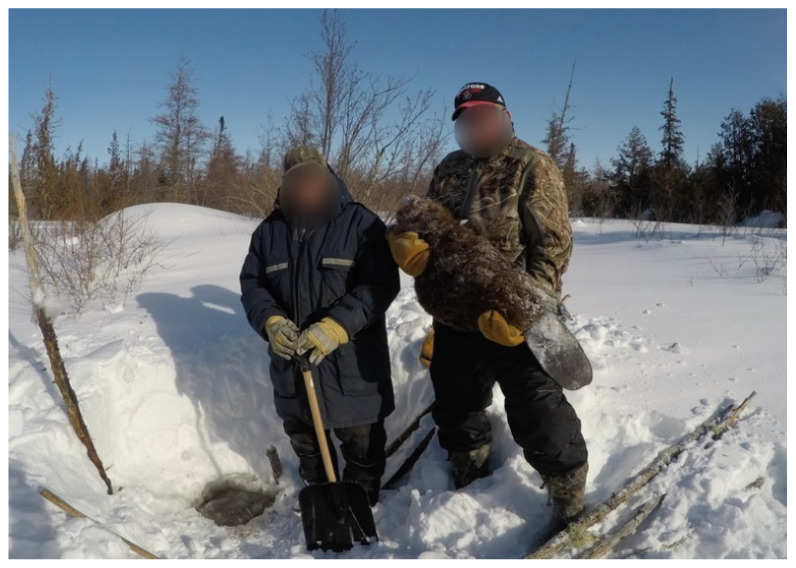
An Elder and on-the-land expert with a large beaver.

Familial and social supports are an integral part of Cree culture. The need for increased interactions with friends, family members, and mentors was identified as an opportunity to share well-being by the participants (Figure 2).

These interactions allow for the transfer of vital intergenerational knowledge. The absence of these important interactions for many of the participants was identified as one of the main reasons for a lack of participation in on-the-land cultural activities prior to this program:

*“I think it’s like, maybe their parents don’t have the experience…I think most people don’t have the equipment to take their family out… At the same time, I think they’re not too confident in themselves to take their family out cause they don’t have that experience”*

(Participant 3, Elder);

*“There’s all education, like Elders you know, to maintain trapping … Because why do young generation today depend on social assistant when you know there’s lots of other opportunities, they can explore you know within their area, you know for income… In the older days, the parents, both parents I’d say they came up in their own time because that was a survival though, and again you know as I adapted, I was there, I seen it all and that’s why I still I still practice a bit*

(Participant 18, Elder).

Some participants also discussed the lack of familial supports as a barrier to their well-being:

*“I just wanna learn these things so I can teach them to my son when he gets older… my father wasn’t around when I was young, I grew up with a stepdad though…I’d like to teach others too… Some of my friends don’t have outdoor experience”*

(Participant 28, Youth);

*“When I was a kid growing up, that was a barrier too, cause I didn’t grow up with a father…I had a mother that struggled… I didn’t have much hunting, and I wanted more as a kid…When I was 13 years a friend invited me, he told his dad, this guy never hunts, but he likes to, he likes to go on the land so, the old guy took me and I’ve been out since”*

(Participant 33, Expert).

Although many of the participants did not have any experience of trapping prior to this program, they used this opportunity to not only gain experience for themselves, but to share it with others as well:

*“I wanna learn how to do those kind of things … learn how to make a trap. Like what I just did… able to do it on my own, you know to have my own four-wheeler… I want my own camp somewhere where my kids can go…To start hunting when they wanna hunt, and like I wanna be able to teach them that you know. I just need to learn first. That’s what’s stopping me”*

(Participant 8, Youth).

The shared sentiments of participants were also about taking care of the Elders and other community members who require assistance, as an important aspect of well-being:

*“My grandparents always taught me that, once you start doing that then don’t forget about the Elders when you’re trapping and hunting, provide food for them so I do that every summer…I did that last summer, I went and set nets for sturgeon, and I grabbed lots of sturgeon, but I just give it to the community”*

(Participant 3, Elder);

*“Just keep myself busy not to think about it, clean up the yard for my family, or go out and get wood, or work with my uncle when he needs something done, help him out, or if anyone else offers me any other jobs”*

(Participant 21, Youth).

Reflecting on the familial and social supports in the context of their experiences, some of the participants discussed how this program not only allowed them to build new relationships, but also to strengthen their existing relationships. When reviewing his photos, one youth participant had photos of him and a friend who participated in the program as well, *“He’s my mentor basically… I look up to him I guess… he teaches me everything… his knowledge will be passed down to my kids”* (Participant 8, Youth). Reviewing the photographs that they took throughout the program invoked many of them to reflect on their personal experiences in the bush. When discussing how he gained his knowledge, one participant stated: *“My father…we were in the bush all the time eh?”* (Participant 12, Expert). Similarly, a youth participant also reflected, stating,

*“Before this I didn’t really have that because when I was younger, I use to go out in the bush with my Mooshum [Grandfather] there and we weren’t beaver hunting or anything we were geese hunting and like hunting for fish and things like that”*

(Participant 16, Youth).

Overall, the program allowed the participants to bridge together and strengthen their familial and social supports and to empower themselves within their community.

**Identity**

Cultural Continuity

Cultural continuity was an important aspect of identity and well-being for many of the participants. Colonization and residential school have caused disruptions in aspects of the *Omushkego* Cree culture. One of those aspects is language, *“We’ve lost so many words…our language is the same except those words are missing now but they’re gone because they’re never spoken so that’s important for us to try to keep our language”* (Participant 3, Elder). For this reason, some of the participants found challenges communicating with some of the Elders and on-the-land experts, as they did not speak high-level Cree as compared to conversational Cree or no Cree at all. This was identified as a barrier to well-being by one participant, who stated, *“I don’t understand it. I was raised in a city by foster parents”* (Participant 8).

Despite some difficulties with communication, the youth participants were taught through observational learning. They expressed a strong relation to their culture through their time on the land:

*“It was important to learn because it’s a part of my culture and I don’t really know how to explain it, but I could just feel myself being out there along with nature…I felt connected…Like I felt, who I’m supposed to be”*

(Participant 16, Youth);

*“One of the young guys talking to me, he was all happy, and he says, I was happy to go out there, he says it was emotional for him too, he was sitting there, and he says you know it’s something I missed when I was growing up…I didn’t have a chance, and but now I learnt…the young guy says, I wanna go out on the land one of these days when I have my own grandchildren, and show them how, what I learnt”*

(Participant 33, Expert).

**Figure 3:**
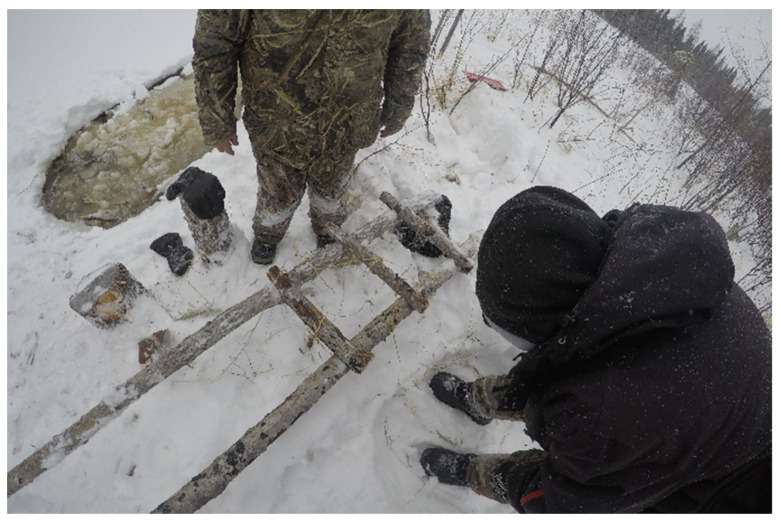
An on-the-land expert showing a youth participant how to set a snare for beaver trapping.

Participants who had exposure to trapping and traditional activities from a young age identified how this has shaped their identities and relationship with culture. Some recalled these memories, while reviewing their footage (Figure 3), *“Its fun doing that eh…doing that since around 15 years old… My father, we were in the bush all the time, eh? We had a camp over there (points to photo)”* (Participant 12, Expert).

Similarly, while looking at a photo, another participant stated, *“I was raised in the bush eh”* (Participant 1, Expert).

By engaging in new experiences, whether beaver trapping for the first time or teaching youth, the participants all contributed to furthering cultural continuity.

Traditional Activities and Food

A common theme in many of the discussions was the importance of the traditional activities and traditional food to well-being. Overall, a decline in participation in traditional on-the-land activities was identified by many of the participants, many of whom had never gone out on the land prior to this program. In this context, Elders and experienced participants discussed the continuity of the program, *“Yeah, the thing is, keep this going, it’s a good thing …what’s been going on there…it’s good to keep it going so the younger generation can take part too”* (Participant 33, Expert). The youth participants equally expressed their excitement for the program, *“I was excited that day to got out in the bush to get experience because I never really went out in the bush to go trap beavers before”* (Participant 16, Youth).

Although this program was centered on the beaver harvest, the participants also discussed other types of traditional activities and how they relate to well-being. These activities, including beaver trapping, all reinforced strong values of resilience. For many of the participants, the challenges faced during their participation forced them to adapt by developing new skills:

*“So, when I was younger, I was taught many things … and that’s one of the things that I’d like to bring back in the community…For people to learn to make snowshoes, learn how to make a birch bark at home. Those things were a long time ago…If you lived on the land, you have to, if you went to live in the bush you had to learn to make your own canoe to come home or toboggans or snowshoes…you have to make the preparation and all that you have learn…just like life skills, eh? But those are things that are learnt over a period of time…You have to spend out on the land to be able to do that…but you’re when you’re young. My grandmother use to teach me, I was just helping them at first eh…I began to learn it on my own…I just watch them when I was younger, but I remember”*

(Participant 3, Elder);

*“It’s better when you get up in the morning, looking forward for the next day, you get ready at night. Get up early and go… the cold doesn’t really stop us, although sometimes it would be 40 or 50 below but we adapt”*

(Participant 13, Expert).

During discussions of well-being, many of the participants discussed the importance of traditional foods:

*“You know for the well-being for say some members to still harvest beaver for income as well like they harvest because of the food…So that’s how I’d define well-being with the community members for their well-being, and healthier foods.”*

(Participant 18, Elder);

*“I also keep some for myself like I put it in my freezer for my winter supply. But then I still give it to the Elders. Sometimes I’ll surprise them… they’ll be happy. There’s lots of people that do that, so there’s lots of food supply for the Elders that are unable to do stuff like that. Because they want to eat that eh? Especially the people at the hospital”*

(Participant 3, Elder).

When discussing cultural well-being, the youth participants shared similar sentiments, *“Trapping, stay around there, stay in the bush and eat wild meat”* (Participant 10, Youth). Similarly, when describing what was important to his well-being, one participant stated, *“Hunting man… bringing home the food you know?”* (Participant 8, Youth).

The dialogues about traditional food centered on cultural connection, and also addressing issues of food security. Due to its geographic location, the food must be flown into the community, causing it to be very expensive. The store-bought foods which are lower in price are generally found to be higher in saturated fats and refined sugar. For the community members experiencing illnesses, such as diabetes and high blood pressure, this creates many challenges. For this reason, many rely on traditional food as a source of nutritious and accessible food,

*“I just want to share that it’s just a good thing to learn. I guess everybody should learn this, not just First Nations… this is a good, a perfect example of maybe one day, I learned to go hunting for my own food…but it’s a good thing to learn, maybe one day there won’t be any food or anything”*

(Participant 16, Youth).

The lack of consumption of traditional foods and the increased consumption of market foods has been raised as a concern by many in the community, who see the shift negatively impacting their health and well-being. There was a consensus amongst the participants that well-being was synonymous with partaking in the traditional activities and the consumption of traditional foods.

**Healing**

Barriers

When discussing the barriers to well-being, participants discussed an array of factors which not only impact their well-being, but also their ability to partake in traditional activities. As stated by one Elder,

*“You start to wear out your body… You know you start to like get arthritis and you start to hurt, but you can’t do nothing about that eh? I went to the hospital once cause I had a bad knee, I have Arthritis on my knee and the doctor asked me if I want medicine? and I said why do I need that, what am I going to do with that? He says its going to stop the pain, and I told him no I don’t want any medicine I think I can tolerate the pain and I can control it and just limit my you know, physical activity not to be too strenuous and learn to give myself time and then if I go out on the land maybe come home and just relax”*

(Participant 3, Elder).

Similarly, another Elder stated, *“When he gets sick, that’s the only time he can’t go out”* (Participant 36, Elder; English Translation from Cree).

When discussing the barriers to well-being, the participants discussed other factors which interfere in their well-being and ability to participate:

*“Yes, there’s a lot stopping. There’s always temptation around. That’s what’s stopping the well-being for me. There are temptations like alcohol…and there’s drug in town…and there is things like negative attention that’s what makes me not stop, no motivation for that to happen”*

(Participant 21, Youth);

*“That’s the big difference… since I started a full-time job, I almost don’t have that much time to spend on the land as I want”*

(Participant 3, Elder).

When comparing his ability to trap to his father’s skill, one participant discussed his barriers, stating, *“Smoking…yeah if I could quit that I could be just as excellent as him”* (Participant 13, Expert). Another participant who experienced a recent loss in his family discussed how going into the bush helped him, *“there’s more life over there…fresh air, fresh water, everything”* (Participant 12, Expert).

Overall, although the participants all identified of the individual barriers which impacted their well-being and ability to take part in on-the-land activities, their sentiments were followed by discussions of resilience and healing.

Physical, Mental, and Emotional Healing

**Figure 4:**
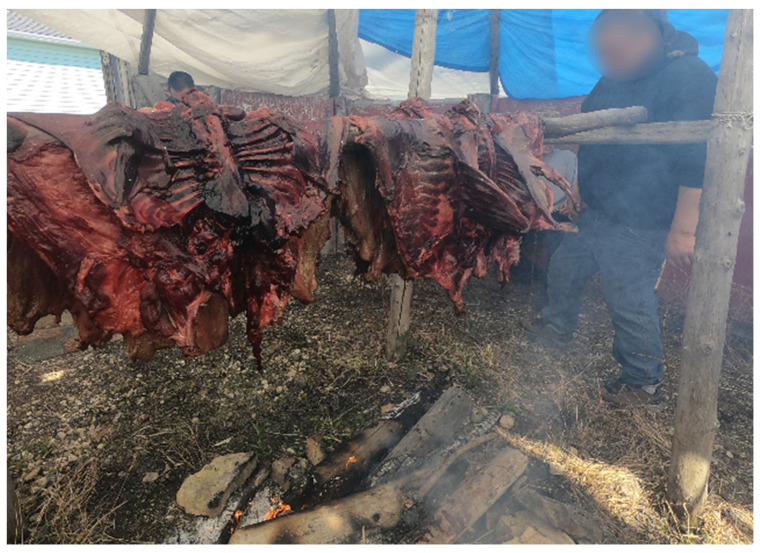
Beaver meat being prepared by an on-the-land expert using traditional methods of smoking.

Within many of the Indigenous teachings, physical, emotional, mental, and spiritual health are all seen as interconnected. Thus, addressing the issues pertaining to physical health cannot be completed without ensuring a balance in all of the other areas. Many participants discussed their time on the land as an opportunity to improve their well-being in many aspects, from physical activity to the increased consumption of traditional food (Figure 4).

For many of the participants, the time spent on the land was medicine,

*“It’s like my uncle says… its good to spend time on the land…you smell all that fresh air and at the same time it’s medicine that you’re inhaling, eh? So, it’s good for your spirit, it’s also good for your mind, it helps you become strong, eh? You’re not weak, because you’re always moving”*

(Participant 3, Elder);

*“For my well-being, well I try not to disturb the environment…the reason I hunt is, I hunt caribous and I hunt moose the majority of the time and that’s my food supply for the year, because it’s healthier, because I’m a diabetic”*

(Participant 18, Elder);

*“He said being outside, trapping and all that, just by being outside and being active…He says it’s very healthy for the breathing…he said you can get sick from being inside all the time…But being out there he says it’s very healthy. He says when he used to get sick at home he would get a cold, sore chest, and everything, being at home, and then he would go out on the land, two three nights out there. He say’s his sickness is gone. It’s like being out there, it’s like medicine”*

(Participant 36, Elder; English Translation from Cree);

*“For my health to keep my blood pressure down and, I feel better, just happier and, that’s my well-being. Yeah, try to get away from stress, stressful situations…For me like going in the bush like it’s always nice to be out there”*

(Participant 33, Expert)

*“To be active… To move around, instead of you know sitting at home and eating”*

(Participant 13, Expert).

The reoccurring theme of time on the land being medicine was also reiterated when discussing the ways to measure well-being. For many of the participants, the discussions centered around the different emotions they feel being out in the bush versus in the community. One participant described, *“I’m more energetic*” (Participant 1, Expert). Others also expressed similar emotions:

*“Yeah, it helps out a lot when you’re out in the bush…you feel more open…your thoughts are just like clear”*

(Participant 21, Youth);

*“I just feel better when I’m out there…clears my mind…not thinking about anything else, it’s just fun being out there”*

(Participant 10, Youth);

*“Nice and calm over there…less stress”*

(Participant 1, Expert);

*“Felt nice being out there too. Being away from all the energy in our community…Being out in the nature…you feel no energy around… feels more peaceful.”*

(Participant 16, Youth);

*“Well-being for me is to make me be better … To feel better is, for me, is like when I go in the land or if I go back in the bush, I feel the like calmness…I feel more comfortable out there… Relaxing, I got nothing over there, there’s no radios, there’s no traffic, no vehicles nothing, it’s just, it’s just real relaxed”*

(Participant 33, Expert).

One Elder who had gone through the experience of the residential school system in his childhood, discussed how he healed from the experience. He shared his experiences with the youth participants and how he was able to overcome some of the challenges he faced:

*“I think you just got to learn to open up … or maybe they carry some hurt or pain that they need to resolve… But at some point, in time if they keep going, they’ll come out of it… You just have to get past it eh…Like I said, if you have something for example from your past, that’s because you’re hanging onto it. So as long as you’re hanging onto it, you’re never going to move forward… but that pain, or that memory, it’s never going to go away, it’ll stay there for as long as you let it. It’s like when I was in residential school, I’ll never forget those things, no matter how hard I try, but the best thing for me to do is just keep moving, and leave them behind, and that’s what I do…I just become stronger as I move forward, but I learned a lot from it. And I can use it as a teaching tool, instead of saying these people did this to me and that, I don’t do that…I had to go down the bottom, and then wake up over there somewhere and then come up, so I’ve come a long way up.”*

(Participant 3, Elder).

It is clear from many of these discussions that partaking in the traditional activities allowed the individuals to reconnect with not only the environment and each other but also with their inner selves, to address all of the aspects of health and well-being simultaneously.

**Land**

Transportation and Equipment

Transportation and equipment were important factors discussed by the participants as impacting their ability to partake in the traditional activities. For some of the participants who did not have previous experience, it caused challenges when going out on the land to participate. This was discussed as a reason for some of the individuals who did not have on-the-land experience:

*“There are barriers, yes, cause for some families, or me, like I don’t have a canoe, and that’s one of the barriers, like you can’t always get into the land if you don’t have, you know the necessities to go out into the land”*

(Participant 33, Expert).

**Figure 5:**
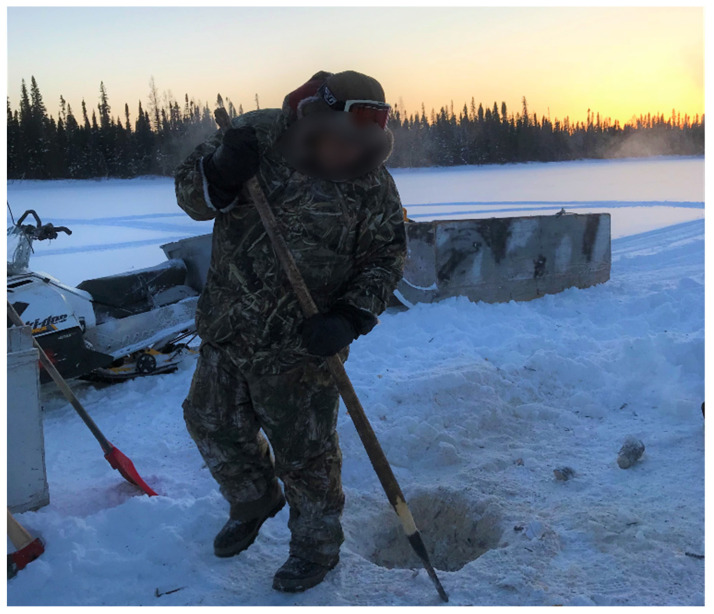
An on-the-land expert using a tool to help clear the ice to set beaver traps.

Similarly, another participant stated, *“I guess having a boat too would be good you know. So, you could just go out there in the bay”* (Participant 8). The beaver trapping (Figure 5) requires a lot of equipment, and, at times, the participants need to travel to remote areas to reach the dams.

Therefore, equipment, such as snowmobiles and gas, are essential. As Fort Albany FN is a remote and isolated community in subarctic Ontario, gas is generally expensive year-round. This proved to be a barrier for some, *“Sometimes, when you want to go you don’t have enough gas”* (Participant 28, Youth). For some who already had experience, they discussed their ability to adapt to create what was needed, *“Lack of tools, but he’s got all his tools. Some of his tools he makes”* (Participant 36, Elder; English Translation from Cree). Similarly, others shared how using your own snowshoes assists when travelling in the bush:

*“You can travel anywhere out on the land, right… we don’t use that much gas and there’s not that much physical work, and then when you get off your machine you sort of walk on the crust or you got to, use snowshoes to go from place to place makes it very easy eh?”*

(Participant 3, Elder);

*“Today they don’t (practice) because the parents, too much modern living… Because for me to purchase a snowmachine I need it for my transportation, and today you purchase a let’s say vehicle, you just use it for your own pleasure, to go in town, you don’t use it for some other use you know that don’t involve wildlife”*

(Participant 18, Elder).

This shift in the cultural practices was observed between those with previous experience and those without, as they were able to adapt to overcome these barriers.

Reconnecting

Through many of the dialogues, it was evident that many of the participants renewed their perceptions of the land and used this space to reflect on their *Omushkego* Cree culture. For some of the participants, the program allowed them to reflect on their experiences with the beaver trapping and other traditional activities:

*“Yeah, it’s very nice… just brings back memories too…when I use to be a little kid when I use to go with my Mooshum out in the bush and this brings back childhood memories”*

(Participant 16, Youth);

*“There’s more well-being if you’re out on the land like camping, like in my past… I used to go in the bush when I was younger, and I was the most comfortable… I feel more happier coming out … I feel relieved”*

(Participant 33, Expert);

“It was a learning experience for me, because I’d seen my dad bring back pelts when I was a child”

(Participant 13, Expert).

**Figure 6:**
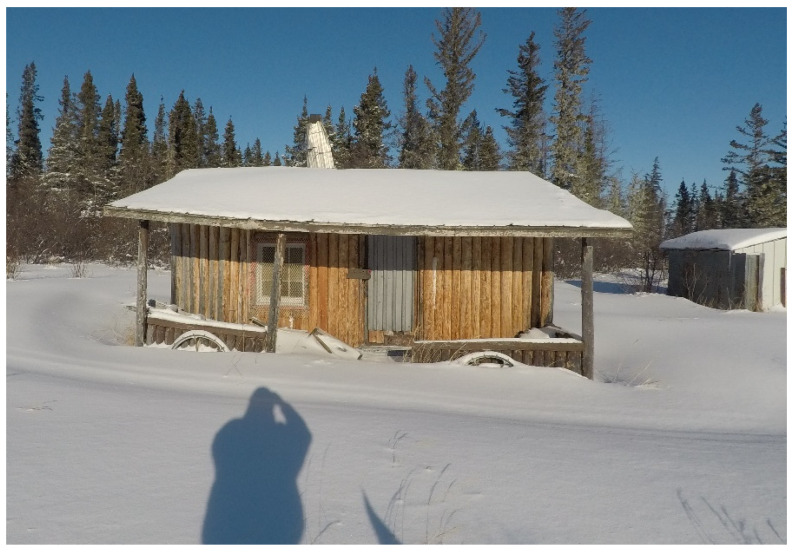
Youth participant taking a photo of a family camp.

When discussing how the land contributes to their well-being, many of the youth participants related it to their identity and described feelings of spirituality and freedom when being out in the bush:

*“That’s actually one of the reasons why that I took pictures of the way it looks right now because I wanted to just show the nature…Cause it feels good being out there. Being with someone. There’s peace out there, and joy out there and a lot of things that you could learn. Cause I learned different things being out there too… made me feel in a spiritual way”*

(Participant 16, Youth) (Figure 6);

*“Instead of having those bad intentions, like the negativity around you, there’s none out there when you’re in the bush, you feel free. Free to do things… Spiritual ways…it’s hard to say like. You can be you out there…you can do whatever you want and without, having any negativity”*

(Participant 21, Youth);

*“When I’m out there, I’m all smiles… I don’t even think about whatever’s going on with me you know…when I was young, I was in (omitted), and we kind of were in like foster home a lot…So I haven’t been outside… But since I moved out here, I liked it…Out there my mind is free to do whatever, to think whatever”*

(Participant 8, Youth);

*“Oh yes, 100% it’s better out there, you go out there, you see everything, you see the land, you see the animals, you know. You also laugh, and you go out there, you talk to someone that you don’t talk to in the community here… it’s surprising how nice these people are. You get to know it’s these guys out in the land more, cause they talk, and they tell you stories …everyone’s sitting there together and is sitting along a fire or wood stove and laughing, and everything everybody seems to be happy out there”*

(Participant 33, Expert).

These shared perspectives reinforce the idea that the land has a positive impact on individuals and provides an opportunity for healing, along with spiritual and cultural continuity.

When discussing cultural well-being, one Elder discussed how the environment represents a belief system for the Indigenous people in Canada, stating,

*“The people there have their own belief system, based on their environment… that’s why we are taught that you know, you should respect everyone’s opinion”*

(Participant 3, Elder).

The well-being of the individuals was also measured through the well-being of the land. Therefore, messages of respect and care were reiterated by the Elders:

*“For me, I respect what I see… Whenever I go out in say the bush, I have to bring all my garbage back you know because I don’t just leave it there …What you can do in the in the summertime is you know, dispose the garbage underground. Better than wintertime you have to bring all your garbage back and put it in the dump … I just respect the land…Land is a life, you don’t kill…You got to respect the environment and if you don’t there will be consequences. You pollute your lakes, what’s going to happen to the beavers? Or what’s going to happen to the fish? Or the birds and the waterfowl? They’ll be carrying diseases and what not because of the disposal, the garbage disposable, so you have to protect it for the well-being”*

(Participant 18, Elder).

The relationships with their land was one of the most important themes, as it was an underlying aspect of every discussion. The interconnectedness with the land, community, and oneself encompasses the various attributes of health and well-being.

#### 3.2.2. Beaver Dam Removal

The major themes identified were: (1) Sharing Knowledge; (2) Cultural Continuity; (3) Healing; and (4) Land.

Prior to the dam removal process, the Elders advised the youth and the expert participants on which of the dams would need to be disassembled to help restore the water flow. When discussing the program, the employee at the water treatment plant gave us his views,

*“I made note to our Band and (omitted), and he told me there was a program here where they were controlling beaver population, and I told them they’re [beavers] wreaking havoc on our creek over here, and they’re constricting [flow] …These beavers were controlled this year, and they haven’t been building any dams now we got no iron-manganese, we got no issues with the water treatment”*

(Participant 37, Water treatment plant employee).

Although these were his preliminary observations, it was important to note that the removal of the dams helped the constricted water flow and addressed some of the community concerns regarding the water quality.

**Sharing Knowledge**

**Figure 7:**
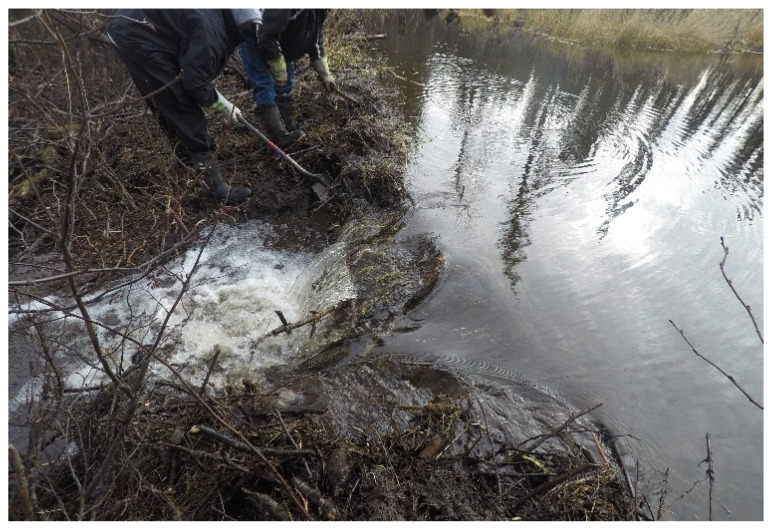
A stream which was one blocked by a beaver dam being cleared by an Elder and youth.

The importance of the knowledge sharing was highlighted during the beaver dam removal program. The knowledge of the environment held by the Elders allowed them to advise on which of the dams needed to be removed to restore the waterways (Figure 7).

This knowledge sharing was described as a means of measuring well-being:

*“I think it can be measured yeah… how much knowledge you can consume in your lifetime, and that knowledge that you have, if you can pass it on to somebody else then it’s something that’s important to attain in your life… you’re responsible to become knowledgeable of your environment so it stays the way you think it should be or the way you were taught. It should be left eh, unharmed or untarnished”*

(Participant 3, Elder).

Not only was this a way to measure the well-being in the community, but also an opportunity to improve the well-being. Participants discussed using situations, such as the environmental concerns within the community, as learning opportunities for the youth:

*“That’s what I would like to see, for the program to continue. It’s a good program I think yeah. I get to see a lot of young people on the land… and they won’t forget, eh? It’ll stay with them, and they’ll just get better at it”*

(Participant 3, Elder);

*“Like going in the bush like it’s always nice to be out there… Being out there with people, walking together, just talking together… it could be talking with the young people, or talking with the older guys and we just, always tell stories together eh, but it’s good”*

(Participant 33, Expert).

Other participants described creating their own moments of learning, *“If I want to learn something, then I’d be with a teacher, like my dad. But if I want to go by myself, I’ll go by myself. I like being out there. It’s nice out there”* (Participant 13, Expert).

These discussions highlight the need for the spaces and opportunities to increase the sharing of the traditional knowledge to help address the imminent environmental and health concerns. The importance of the familial and social supports was observed in these discussions to help the continuity of the knowledge sharing:

*“You know, when the birds come back from down south, they migrate here, and they nest, and this is where they live. They’re not strangers, eh? They’re just going on vacation I guess (laughs), and they come back in the spring right? So, when they come back here their mom just teaches the young how to survive and how to behave and then when the parent leaves, he takes the young and he guides them to where they’re going for winter eh? and when they come back that same mom brings back the same the same little birds eh? Guides them back home, and after that…her job is done and they separate from their young and so it doesn’t matter if the if the mother isn’t there anymore, those little birds that were with their mom had to learn how to live long enough to understand. How to survive and they know how to get over there and back and they’ll do the same thing to their offspring, and that’s just our teachings and that’s how our culture is”*

(Participant 3, Elder).

Overall, the participation in this program allowed for the creation of a space to share knowledge and create the important connections for more familial and social supports for the individuals.

**Cultural Continuity**

Another theme centered around the impacts of colonization and the residential school system; how these events resulted in a shift away from the *Omushkego* Cree culture. Many of the participants expressed the need for programs based on the *Omushkego* Cree traditions and values, for the renewal of cultural continuity:

*“Well, the only thing about this project is that I would like to see it continue, but I would like to see it continue in the way we lived our life… we lived every season in a different way…. Those things are what’s missing now in our community, we don’t practice our traditional way of life… I think that’s important for the young people to see so that they can continue… So as long, as there’s nobody doing it and our Elders are getting too old to teach it, but sometimes, opportunity knocks on our door, like this program…They’ll say what of your young people, what are you going to be in the future if you don’t if you don’t pass on knowledge, what are what are they going to be? So how do you answer that question? Best thing is just to try and find ways to teach them”*

(Participant 3, Elder).

Within the community, the meaning of cultural well-being differs between the individuals because of their experiences of the residential schools, and colonization. One Elder discussed these differences, stating:

*“For example, like people have different beliefs…Well-being for one person might be doing cultural activities like going to a sweat and stuff like that, and maybe another person might be just like may be Christian or some other form of religion that they believe in, so they go to church and pray and in the different things like that”*

(Participant 3, Elder).

Another barrier to furthering the cultural continuity is related to the economic issues. Essential items, such as traps and gas for transportation, are expensive to purchase and create barriers for participation:

*“Maybe having more programs like this… If they had it every year then people would save money…There’s a lot of low-income families that can’t buy traps or buy gas fuel”*

(Participant 33, Expert);

*“Sometimes we have to make our own tools, or, like we’re in isolated areas, there’s not much to do here, I mean there is much to do here it’s just that the barriers would be, not to have these things because they’ll be very expensive to have”*

(Participant 13, Expert).

**Figure 8:**
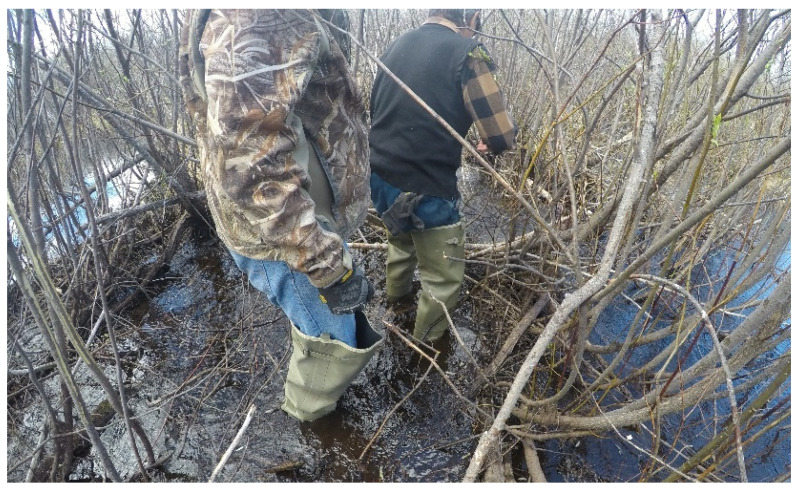
An Elder and On-the-land expert participant using hip waders to access a dam.

The participants also expressed the inability to access certain areas in the bush (Figure 8) because of the climate, “*There are places you can’t go after freeze up…If there were trails, other trails that go into the land”* (Participant 33, Expert).

The idea of culture in these contexts is synonymous with identity. For many of the participants, their life experience has defined what well-being is for them. When discussing why it was important for him to take part in these traditional activities, he identified himself, *“As a trapper’s son*” (Participant 13, Expert).

For one participant, when discussing culture and its impacts on well-being, he discussed that it was important to him. Similarly, another participant stated, *“Just feels good…Just like to go out hunting”* (Participant 11, Youth). Within the community, the cultural continuity is rooted in resilience, therefore, impacting well-being on an individual and community level.

**Healing**

The participants discussed the interconnected aspects of health and well-being, with healing being a prominent theme. For some of the participants, their physical health was a barrier to participation in traditional activities: *“Some barriers could be like health wise, we won’t be able to go out when we’re sick especially long-term illness*” (Participant 36, Elder; English Translation from Cree).

Other participants expressed how taking part in the removal of the dams allowed them to increase their levels of physical activity, directly impacting their health and well-being:

*“Well-being like what we did there was going out that was about half an hour walk to get there. To keep active so I can do the, whatever the community needs or what I need to do for myself, for my camp or whatever”*

(Participant 13, Expert);

*“I’m more healthier out there, my blood pressures down”*

(Participant 33, Expert).

Similarly, participants described the impacts of other traditional activities on well-being:

*“Exercise, I guess yea, It’s good walking around the bush, eh? To be healthy”*

(Participant 12, Expert);

*“By keeping active, going out on the land …Even on my days off, I still go out in the bush to do my woodwork, many things like that. There is a lot to do out there”*

(Participant 13, Expert).

**Figure 9:**
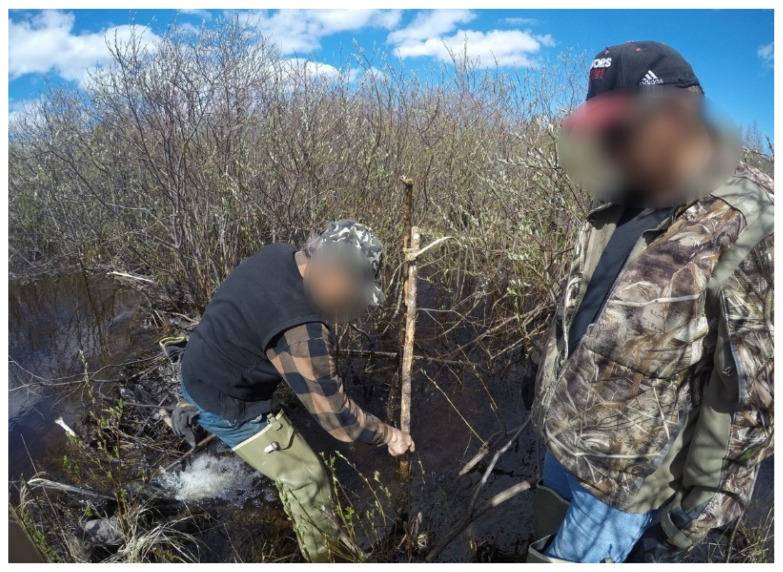
An Elder and on-the-land expert setting a beaver trap prior to removing the dam.

The time spent on the land was seen as an opportunity for healing, as it provided a space for knowledge transfer and increased engagement amongst the community members (Figure 9),

*“Like this program here, for young people it’s an opportunity for them to work with people like me, like people that were in their shoes… That’s what I tell people. I was like you once, but I had to work hard to get better, and you got to do the same thing. It’s a lot of work. It’s not easy. It’s a lot of work to walk that healing journey… and there’s people you got to talk to… I guess some of my friends are on the same path so, we always connect, eh? Every once in a while.”*

(Participant 3, Elder).

Although many of the benefits were recognized, the participants also recognized the trauma and other barriers which were stopping them from participating in the on-the-land activities prior to this program,

*“Well, we have right now is a community crisis, a drug crisis. If that was gone, a lot, everything would be back to normal. Like before… the alcohol was alright but the drugs now. Oh my god it’s like nobody wants to go out, all they want to do is get high. But once the drugs are gone, everybody’s going to go back out again. I’ve seen it before. A lot of people were out there, a lot of young people were out there. Out in the bay, up the river, on the land, in the swamp, but when the drugs come around, nobody goes out anymore really. There’s only a few, but it’s not much.”*

(Participant 13, Expert);

*“I went through lots in my younger days…I was very defiant because I was always controlled in a residential school, so when I came out, I think I felt I was free so I could do anything. It didn’t matter what I did, so I saw authority as a threat. I was defiant… I got in a lot of trouble for that (laughs)…But I learnt, and I began to understand… over a period of time. I think when people started opening up, then I began to understand, because sometimes I thought some of the things I remember were just dreams, but they were real, I think in my mind I just locked them away and then somebody else opened up and then mine woke up, and then those were the hardest times of my life, when I had to really come to terms that what I thought were dreams were real, something that happened to me…when I was younger… there are a lot of people out there, like Elders for example, who were the ones that were mostly very helpful to me… I spent most of my time talking with my Elders and telling them what I was going through, and they would tell me that, as long as you hang onto that, you’ll never get better. You have to learn to let go… and they said to me. How do I do that? And he said you have to forgive yourself first, because if you can’t forgive yourself first, you’ll never forgive the people that hurt you, so I had to do that first, and that took a long time it’s not easy…so now when I don’t carry any grudges right? I just live my life. It’s my life, and I’m the one in control here”*

(Participant 3, Elder).

The lessons learned from each other, and the time spent reflecting on one’s personal experiences, illustrate the importance of the land and traditional activity for health and well-being.

**Land**

In every theme presented, the connection with the land was an underlying factor which connected all of the discussions and participants. It was a space for cultural continuity, the establishment of one’s identity, where healing took place, and the exchange of knowledge. Participation in this program encompassed many values, including communication, working together, adaption, and sharing. One Elder spoke of these values:

*“Well-being can be when you are outdoors and you’re with other people, you can share stories … You’re more inclined to share things when you’re out on the land, eh? As opposed to if you’re sitting in a room or in a house there’s a lot of distractions … you walk into an airport and there’s no communication, there’s just people (phone gesture) screen time, eh? But if you’re in the bush you can’t, cause there’s no internet… so you’re forced to look at the person eye to eye, and you talk to each other, look at each other so that’s something for me that’s important …the most important is that you should be able to communicate as a person with another human being through just talking or sharing things that you’ve learned or if you don’t understand to ask questions that way, that’s well-being for me”*

(Participant 3, Elder).

When asked whether the land impacts their well-being, many of the participants expressed positive emotions:

*“I like nature, I like the beauty of it. There is beauty you can see it…and when you’re out there walking around, air is clean. It just feels so peaceful out there”*

(Participant 13, Expert);

*“Well-being is, for me I guess, going out on the land there…being happy*

(Participant 33, Expert);

*“When I’m out there, I feel like I’m where I’m supposed to be… I can hear things, I hear the trees… and when I hear them, I remember all the things that my Elders, like the stories they told me…I remember my Elders that have passed away and their advice…It’s like regenerating me and helping me to become a good person”*

(Participant 3, Elder).

**Figure 10:**
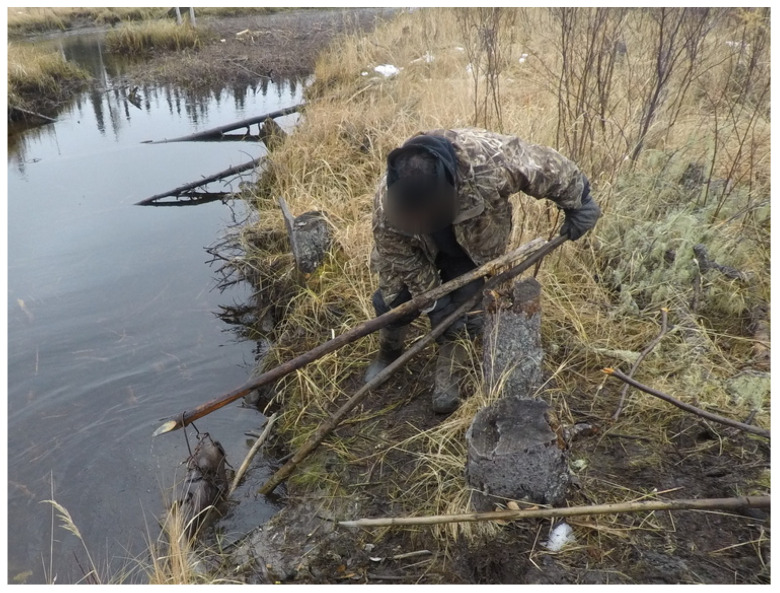
An on-the-land expert removing a beaver which was caught using a conibear trap.

Taking part in the removal of the beaver dams project provided an opportunity for the participants to use their knowledge and experience to address an issue which was impacting the community (Figure 10). For many of the participants, this was a reminder of what their Elders taught them,

*“I believe you should leave the land the way it is, because that’s how we were taught. You know everything that we know. Our ancestors taught us as best as they can, you know. To teach us to respect the land, because they know that as long as we show our respect to it, it will continue to provide for us and then the generations after, that are yet to be born”*

(Participant 3, Elder).

It was evident through the discussions that the land was central to the health and well-being of the participants. The land was a source of resilience, but also a way to reconnect with ancestors and provide for the future generations. Through their participation in this program, individuals furthered their cultural continuity, allowing for the healing of the land and themselves.

## 4. Discussion

The *Amisk* (Beaver) program helped reconnect the youth with their traditional homeland, their Elders, and cultural traditions, to address community concerns of local flooding, and improve the water quality in the lake that provides the community with drinking water. Additionally, using a two-eyed seeing approach allowed us to improve our understanding of stress and well-being from both the biomedical and the First Nations’ perspectives.

### 4.1. Biomedical Perspective of Stress: Cortisol Levels

The results of our analysis provide us with some initial insight into how the trapping and the removal of beaver dams affects the short-term cortisol concentrations (e.g., stress response). From a simplified viewpoint, it would be assumed that cortisol levels would decrease post-participation in the activities, because being on the land has often been described as being relaxing and important for Indigenous’ well-being. However, no significant changes were observed in the participants post-trapping, and a statistically significant increase was observed post-removal of the beaver dams. Perhaps the vigorous physical activity needed for the removal of the beaver dams was responsible for the significantly elevated cortisol levels post-participation. Indeed, it has been shown that the participants in on-the-land programs were significantly more physically active than people who spent less time on the land [58]. Factors, such as aerobic activity, have been shown to elicit acute increases in cortisol [54,59]. As described by many of the participants, the beaver harvesting and dam removal activities were both physically demanding, which was physically beneficial, especially for the Elders and those who suffered from health conditions, such as diabetes.

The HPA axis, which drives production of cortisol to increase energy (e.g., raising blood glucose levels), responds to a variety of physiological and psychological situations, such as waking up and physical exercise, which do not necessarily give rise to a negative effect [38,43,60,61]. Therefore, it is important to note that there are a number of situational factors, such as the familiarity of the task or the resulting “achievement”, which impact the effect of cortisol [58]. Within the literature, there are few studies measuring the pre- and post-intervention salivary cortisol concentrations in Indigenous populations. One study in Bolivia observed the cortisol responses in Indigenous Tsimane subsistence hunters and observed an increase in cortisol and testosterone concentrations at the time of the kill and once they returned home, with a decrease for those who were unsuccessful [59].

In our program, the trapped beavers were immediately frozen and processed later, as per the advice of the Elders, as the smoking and skinning process was completed at the end of the trapping program to provide an additional learning opportunity for more of the youth to participate. This may be one reason as to why there was no significant response observed post-participation in trapping, as there was no immediate “achievement” stimulating the release of cortisol [60], but there would be a feeling of accomplishment in providing food for their families and others [59]. This viewpoint was apparent in the present study during interviews with many of the youths, experienced participants, and the Elders, who stated that getting food and sharing it with others was an important part of the *Omushkego* Cree culture.

For the removal of the beaver dams, participants described having a responsibility from their ancestors to protect and care for the land, not only for the current, but for also the future generations. Therefore, the removal of the beaver dams provided an immediate feeling of “achievement” for participants, as their efforts helped to restore the water flow in the restricted areas which were causing flooding. This positive feedback on what was a challenging task has been observed to have a higher cortisol response than to those with no or negative feedback [62]. These results provide us with preliminary insight into the impacts of traditional harvesting behavior of men in subsistence societies, where acute cortisol levels are seen to increase as an evolutionary trait related to familial provisions and land stewardship [59].

Another factor which should be considered, when addressing stress in Indigenous populations using salivary cortisol, is acculturative stress, allostatic load [63], and blunted cortisol awakening response (CAR) [42]. Acculturative stress is the reduction in health status for those who have experienced acculturation [3], that is, changes to the Indigenous culture as a result of colonization and the assimilative efforts of colonizers [63]. Assimilative efforts in Canada, such as legal assimilation (voluntary and involuntary), the residential school system, the “sixties scoop”, and environmental assimilation, have transgenerational impacts [2,3,64,65]. This was discussed by one of the Elders, who discussed the negative impacts that colonization had on youth, who were unable to experience on-the-land activities with their families. The biological impacts of acculturative stress throughout generations has resulted in a greater allostatic load on the body [1,64,66], which refers to chronic altered cortisol concentrations, due to the cumulative burdens of chronic stress [1]. Several studies have shown that Indigenous populations experience more stressful life events compared to other non-Indigenous populations [1,3,36,38,42]. As stated by Sarnyai, et al. [66], the varying degrees of allostatic load have direct impacts on cognitive and physical functioning and can be used as a potential measure of the cumulative biological burden. This has also been observed as an underlying factor for many health disparities, such as diabetes, hypertension, and poorer mental health [42,66]. In a study by Chief Moon-Riley, et al. [64], it was suggested that the colonial residential school experiences have been embedded and passed down through generations, resulting in the dysregulation of the allostatic systems of maternal residential school survivors. Many of the participants either had parents that were in residential school or attended themselves. Other participants discussed factors such as the early loss of family members [67], substance abuse [68], and spending early years in the foster care system [69], all which have been shown to elevate the allostatic load. Similar results were observed in a study by Thayer, et al. [70], who found that early life trauma in Native American adults was related to post-traumatic stress disorder (PTSD), and elevated allostatic load in adulthood, irrespective of substance abuse or other traumatic exposures in adulthood. Additionally, a blunted or flattened CAR is commonly seen in those who have experienced childhood adversity, such as residential school [64], or experienced severe mental health disorders, such as depression or PTSD [71,72]. In other words, the typical cortisol release pattern, that follows a circadian rhythm characterized by cortisol secretion peaking between 30 to 45 min post awakening [73], becomes replaced by a blunted CAR, where cortisol release has been attenuated, within the first 30 min [42]. Adding further, in a study by Berger, Leicht, Slatcher, Kraeuter, Ketheesan, Larkins and Sarnyai [42] it was observed that the circadian cortisol pattern was significantly different between the Indigenous and non-Indigenous participants, with the Indigenous participants showing a significantly flatter CAR. Studies have shown a high heritability of CAR, suggesting that genetic factors substantially influence the HPA axis function [42,74]. This is of particular importance to Indigenous populations, due to the widely recognized concept of trans-generational transmission of trauma, which is the transmission of vulnerability to PTSD from one affected individual to the next generation [3,42,64]. Therefore, it is imperative to acknowledge that, when assessing cortisol responses, exposures to childhood adversity, PTSD and trans-generational transmission of trauma does impact the individual profiles of the participants.

Lastly, although our results potentially infer that the removal of the beaver dams significantly increased the participants’ cortisol concentrations post-activity, all of the participants mentioned their positive experiences with all of the aspects of both the beaver harvesting and beaver dam removal activities, and how participating in the beaver program contributed to both individual and community well-being. There is an increasing awareness that health and well-being are more than just biomedical constructs, and must also be viewed through social and social–cultural lenses [32]. This type of perspective with respect to health and well-being is relatively new in western scientific research, however, it has always been a part of Indigenous teachings.

### 4.2. A First Nations’ Perspective on Well-Being

Cultural identity relating to well-being has been a longstanding value embedded in Indigenous teachings, such as the medicine wheel, where the alignment of internal and external factors is required to achieve well-being [5,75]. The results of our study presented a similar perspective, with many of the participants, especially the Elders, speaking of the direct correlation between the health of the land, and the overall well-being of the community, and their own personal well-being. These results are in alignment with the often-stated position that land-centered programs/interventions positively impact on human health and well-being. On an individual level, the participants discussed the need for cultural engagement through activities, such as trapping, to provide them with access to traditional food, to obtain and share traditional knowledge, and to improve community supports. Our results were similar to a systematic review assessing the impacts of traditional ceremonial activities on Indigenous adults, where benefits at the physical, spiritual, mental, emotional, and community level were reported [76]. In the present study, when participants were on the land, many of the participants stated they felt they were who they were actually supposed to be, or experienced feelings of calmness, freedom, and relaxation. Participants frequently described the land as medicine and healing. Another recent systematic review by Akbar, et al. [77] examining Indigenous youth in Canada, USA, New Zealand, and Australia, reported that taking part in traditional physical activities were affected by familial and communal relationships and described numerous emotional and spiritual benefits. Similar results have been noted in other studies assessing the impacts of land-based activities on diabetes [78,79,80], cancer [80], cardiovascular health [81,82], wellness [83,84], and well-being [85]. This idea of Indigenous culture, identity, lifestyles, well-being, and being deeply rooted in their homelands, has recently been acknowledged in health and research [4,26,83,86], but has long been espoused by Indigenous leadership and organizations [2,65]. Thus, it is not surprising that the land is of central importance to the *Omushkego* Cree culture, where reciprocal relationships with the land provide for their subsistence lifestyles, and in return they are stewards of the land [65,87,88]. In a study by Hossain and Lamb [5], it was observed that the promotion of and support for traditional activities, such as hunting, led to greater psychological well-being for Indigenous people in Canada living in non-metropolitan areas. As there is a great heterogeneity in Indigenous traditions, it was suggested that different approaches may be needed to address Indigenous well-being, depending on the location [5].

Maintaining Indigenous “management” practices may be necessary for the sustainability of the environment, as has been recently emphasized [89,90,91]. The practices such as trapping, which provide a source of physical activity and nutritious food, may help to maintain the environment through Indigenous knowledge [26,35,86]. Indigenous knowledge refers to the Indigenous values, benefits, and practices which have been accrued over generations for the conservation and protection of the land [14,25,28]. Through their knowledge that has been passed down through generations, and their connection with the land, the Indigenous communities and/or certain individuals are seen as knowledge holders. In the present study, the Elders guided which of the dams needed to be removed to restore the waterways to a more natural state to alleviate community flooding. Similarly, Indigenous knowledge has been increasingly used to identify the complex interactions between climate change and northern communities [25,75,92].

Although there has been a disruption to the dissemination of Indigenous knowledge because of the residential school system [24], and other assimilative initiatives in Canada and worldwide [2,65], on-the-land programs are essential to cultural continuity, environmental sustainability, and well-being. The complementary use of Indigenous and mainstream western knowledge systems has been identified as an inclusive and equitable way for managing ecosystems and addressing environmental challenges [93]. The role of Indigenous people in sustainable land management is prevalent in Australia, where Indigenous land and sea management programs (ILSMPs) have been shown to play an important role in well-being, providing a range of cultural, social, and environmental benefits [94,95,96]. The ILSMPs further biodiversity conservation, protect natural resources and heritage, while also creating employment and economic opportunities [96]. A study by Larson, Stoeckl, Jarvis, Addison, Prior and Esparon [95] evaluating the well-being impacts on the Ewamian people involved in ILSMPs in north Queensland, Australia, found that having legal rights and access to “country” (i.e., land, sea, air), knowing that the country is being looking after, and having strong cultural connections were all identified as important factors to well-being. It was suggested that these factors offered insights into the development of aid programs, environmental initiatives, and educational programs [95]. These sentiments were similar to those expressed by the participants in our program who reiterated the connections between the land and culture, land being important to well-being, as well as taking care of the environment out of respect for their ancestors, as well as current and future generations. Prior to our program, the declining engagement of youths with the Elders was reported by the Elders and on-the-land experts as a factor contributing to the decline of Indigenous knowledge-sharing in the community. Therefore, it is imperative that programs such as ours, created for knowledge sharing, are designed to not only build relations, but also to support communication across the barriers of language, experience, or other world views [93].

The reported loss of Indigenous knowledge [97] has been further accelerated by technological advances which have led to decreased interactions with the Elders and have disrupted familial and community trapping and harvesting practices [5,24,98]. Many of the participants attributed their lack of experience to the absence of knowledgeable family members, because of the impacts of the residential school system and other assimilative initiatives. This was similar to observations in a study by Minkin, Whitelaw, McCarthy and Tsuji [4], where the participants expressed the importance of rebuilding traditional knowledge to increase understanding of the current issues. Another one of the challenges identified by the youth participants in our program was communication barriers, as many of the Elders and on-the-land experts spoke Cree. Declining use of the Cree language within communities has been identified as an erosion of the deeper meanings of connection to land, resulting in a loss of traditional knowledge [3,6,24,99]. Several studies have suggested that a disconnect from Indigenous culture stemming from colonization has been a source of comparatively poorer physical and mental health, and overall lower levels of well-being, in Indigenous peoples worldwide [1,3,5,79,100,101]. Another challenge identified by the youth participants in our study was the lack of equipment to partake in traditional activities, because of the high costs and availability. Although many of the Elders also faced this challenge, they were able to make equipment such as canoes and snowshoes to overcome such barriers. A study by Restoule, Gruner and Metatawabin [6] stressed the importance of bringing Elders and youth together, by creating spaces through the traditional activities, to foster meaningful intergenerational dialogue. Not only do these dialogues help with overcoming the barriers to activities, but they also help to address the growing issue of food security in many communities [8,12].

Many participants expressed the need to consume traditional food; traditional foods are generally considered more nutritious, with game meats and fish having significantly different amino acid profiles compared to market-bought-processed foods [102]. These sentiments were common amongst youth participants, who described the need to provide food for their family, other members of the community, and themselves. Traditional foods have been acknowledged as an important part of cultural continuity and contributing to many aspects of health and well-being [10,80]. In a study by Hanemaayer, Anderson, Haines, Lickers, Lickers Xavier, Gordon and Tait Neufeld [10], youth from a Haudenosaunee community identified families and communities as an important aspect of their perceptions of traditional foods. The benefits of these traditional foods were described by many of the participants in our study, especially those who were diabetic, had high blood pressure, or other health issues which benefited from more physical activity. These types of benefits were reported in a study by Moriarity, Zuk, Liberda and Tsuji [58] that assessed the health measures of *Eeyouch* (Cree) hunters and trappers of the eastern James Bay region of northern Quebec, Canada, participating in the longest running on-the-land program in the world. They found that the participants had higher levels of vigorous and moderate activity per week and higher concentrations of omega-3 polyunsaturated fatty acids (a proxy for traditional food consumption), compared to those who spent less time on the land. As the rates of diabetes and cardiovascular disease are increasing in the Indigenous communities, there is a need for more awareness, particularly amongst the younger populations [6]. In a study by Restoule, Gruner and Metatawabin [6] they identified that being on the land and learning from a place beyond institutional walls, with the support of the Elders, provided a return to traditional Mushkegowuk modes of teaching and learning. Likewise, the Elders in our program echoed similar sentiments, stating the need for more spaces and opportunities for engagement with youth and for more culturally relevant programs to address health and well-being, and further cultural continuity.

The overall success of this program was attributed to the Elders, on-the-land experts, youth, community members, and the Band council who helped to make this possible to address the various community-identified concerns. The Elders who led the program used their knowledge and experience to help guide the program to address the environmental and community-level health and well-being issues. They reiterated their approval of the program through their interviews, and that it should continue using *Omushkego* Cree traditions and values. Utilizing methods, such as the CBPR and/or the two-eyed seeing approach, to engage the expertise of Indigenous knowledge holders during other health interventions and programs was shown to be fundamental to the success of the programs [34,103,104]. Although in the past, research and health models focused on “outside-expert” approaches, it is now being acknowledged that these approaches are poorly suited to facilitate the improved health and well-being status amongst Indigenous communities [34,104,105]. Methods such as the CBPR allow for the inclusion of guidance from community representatives at every stage, break down the barriers between researchers and participants, and ensure cultural relevance [48,103]. Other studies examining the on-the-land activities and the impacts on Indigenous youth, their families, and communities stressed the importance of working with Indigenous communities to incorporate the localized strengths and resources of the Indigenous communities themselves [32]. Another approach to leveraging these strengths and amplifying community voices is through photovoice. In our study, this method allowed for participants to tell their own stories and share their experiences through visuals. Reviewing these visuals also seemingly invoked positive emotions for many of the participants who began to tell stories of their interactions with other participants and their overall experiences. For similar reasons, other studies have used photovoice to address the health and well-being inequities faced by Indigenous communities [10,34,99,106,107]. A study by Carroll, Garroutte, Noonan and Buchwald [106] used photovoice to understand the application of ancestral teachings relating to environmental health and tribal land-use policies in contemporary contexts with a Cherokee community in Oklahoma. They concluded that photovoice was an effective method to communicate the Elders’ environmental knowledge and perspectives. Similarly, another study by Goodman, Snyder, Wilson and Whitford [99], used photovoice to understand the health experiences of urban Indigenous youth, as it was viewed as culturally appropriate and positioned Indigenous knowledge and values at the center of the research. Using integrative approaches such as these and the two-eyed seeing approach, allow for more decolonized approaches to health and well-being, as they place greater value on Indigenous knowledge systems and methods, address power inequities, and promote an equitable and collaborative approach to research with Indigenous peoples [46,108,109]. Incorporating these approaches, along with local expertise, into current and future on-the-land programs is crucial to addressing Indigenous well-being and environmental sustainability.

### 4.3. Transferability

The *Amisk* (beaver) program was specific to Fort Albany FN, Ontario, Canada, to address community-specific issues of a decline in participation in traditional activities by youth, and an overabundance of beavers. Nonetheless, although these issues were specific to this region, there is concern globally for the need to manage overabundant and invasive species as they negatively impact their own and other habitats, and human health [12].

As suggested in a study by Tsuji, Tsuji, Zuk, Davey and Liberda [12], the use of Indigenous harvesting programs in other parts of the world can address not only issues of overabundant and invasive species, but also issues of food security. In Northern Australia, Asian swamp buffalo (*Bubalus bubalis*) were causing damage to the environment, such as saltwater intrusion into sensitive wetlands through the trampling of vegetation, and browsing on remnant monsoon forests during the late dry season when the quality and quantity was at a decline [110]. The Indigenous harvesting practices utilized the buffalo for hides and meat, and, through ecological modelling, it was determined that the harvesting rates would need to increase to alleviate the ecological damage [110]. Likewise, the potential use of an invasive fish species known as the “Asian Carp” as a food source in the USA [111] and China [112] has been reported. As suggested by Li, Prinyawiwatkul, Tan, Luo and Hong [112], opportunities for harvesting the species from the Mississippi River and potentially exporting them to China, where it is considered a delicacy, would provide a mutually beneficial solution providing economic benefit, addressing issues of food security, and protecting the river from further degradation. Similarly, in a study by Morgan and Ho [111], it was determined that the Asian carp was an underutilized food source, due to the negative perceptions from anglers in Missouri. However, it was determined that a change in messaging aimed to promote its great nutritional benefits of omega-3 fatty acids, high protein content and price, along with the potential for population control amongst the species, would allow for both environmental and human health benefits [112].

Another main objective of the present study was to reconnect the youth with the land through traditional trapping practices. Similar studies have been conducted in Australia and the USA [113], and can be employed using culturally-relevant methods in other Indigenous communities worldwide. In Minnesota, the *Ode’imin Giizis* program sought to empower the Indigenous youth through cultural teachings, to address high rates of obesity and issues of food insecurity and access [113]. Children and their families planted and maintained gardens, and were provided with cooking lessons using traditional foods, which ultimately increased the positive health food perceptions and allowed for further development of cultural identity and pride [113]. In Australia, a study by Crowe, et al. [114] found cultural practices, such as fishing and collecting bush tucker (i.e., food from the land, sea, or air), when embedded within the everyday life of Indigenous youth, were associated with healthier lifestyle behaviors.

A community-based participatory research approach to develop culturally relevant programs and a two-eyed seeing approach to evaluate the programs can be modified for other environmental and health issues in Indigenous communities worldwide. Using these types of approaches allows for the inclusion of valuable community perspectives and knowledge, along with ensuring that the research is adhering to the community protocols. To ensure equitable information and research management, our program utilized the First Nations’ principles of OCAP^®^ to guide our research [45]. Although some of the ethical protocols are specific to working with Indigenous communities in Canada, globally there are more protocols being developed for ethical research with Indigenous populations, such as *Te Ara Tika,* a Kaupapa Māori ethical framework which ensures acceptable, accountable, and responsible research which takes into account the history of Māori experiences [115].

### 4.4. Limitations

Our sample size for both of the programs was limited, because the number of Elders and experienced people with the requisite knowledge was small. Further, participation for the beaver dam removal project was drastically decreased, because some of the participants from the beaver harvesting activity portion of the program were required to leave town and relocate due to flooding. Future studies employing more participants would allow us to account for other factors which impact cortisol concentrations, such as age and allostatic load [1,64]. As our goal of the pilot project was to examine changes pre- and post-participation, we believe that this did not unduly influence our results. However, the findings of our cortisol analysis should be interpreted with caution, due to the limited sample sizes.

Samples were only taken once pre- and post-participation in the activity. Therefore, we were unable to measure important factors, such as CAR. Although the cortisol samples were collected at the same time during sampling, to account for the known diurnal cycle known for cortisol, studies have suggested that traditional activities and ceremonies within Indigenous communities should be viewed in their entirety as one cycle [116]. The study by Snodgrass, Most and Upadhyay [116], analyzed changes over a nine-day ritual, where the cortisol concentrations were higher in the last three days of the cycle. It was suggested that the entire ritual was divided into an “earlier” and “later” portion by the participants, explaining the increase in cortisol concentrations towards the end [116]. The results from these studies demonstrate the need to factor in the histories and traditions when conducting population research with Indigenous communities.

Another limitation of this study was that some of the participants only spoke “high” Cree; thus, some of the words and concepts were not directly translatable to the English language. We attempted to overcome this barrier by allowing participants to bring their own translator, who spoke both “high” Cree and English. In the article, the quotes which were translated were labelled “English Translation from Cree”. Lastly, due to the COVID-19 pandemic, several face-to-face meetings had to be cancelled with remote communication being used as the alternative.

## 5. Conclusions

The *Amisk* (beaver) harvesting program allowed the *Omushkego* Cree to take part in traditional on-the-land activities and to engage with youth to revitalize the harvesting practices. By harvesting the overabundant beavers, the participants played an integral role in addressing issues of local flooding, water quality, preserving Indigenous knowledge systems, and concomitantly increased their own and community well-being. As harvesting has a historic significance in the community, the beaver harvesting program allowed participants to reconnect with an important aspect of their Cree identity and ultimately to further cultural continuity.

Taking into account that the biomedical approach of utilizing cortisol to evaluate stress has been successfully used in many non-Indigenous studies [36,43,54,55,60], the importance of an Indigenous perspective cannot be overemphasized with respect to well-being. Indeed, the Indigenous perspective identified many positive benefits of taking part in traditional harvesting practices that would not have been elucidated upon if we just utilized a biomedical approach. The participant perspectives reinforced the importance of trapping practices to share knowledge, as a source of healing, and to reconnect with their identity and the land. Utilizing Indigenous and biomedical perspectives can aid in the development of future on-the-land well-being programs, including ones with environmental sustainability components. For Indigenous peoples worldwide, land-centered programs can be viewed as potential vehicles that can lead to empowerment, increased community social learning, and the improvement of Indigenous well-being.

## Figures and Tables

**Table 1:** Salivary Cortisol (nmol/L) Descriptives for Pre- and Post-Participation in Activities.

Project	Sample	Descriptives	
		n	Mean ± Standard Deviation (SD)
Beaver	Pre	14	12.06 ± 8.17
	Post	14	7.91 ± 5.85
Beaver Dam Breaking	Pre	5	4.32 ± 2.65
	Post	5	12.76 ± 5.11

## Data Availability

Not applicable.

## Appendix A

**Table A1:** The following quotations were not included in the primary text; however, they provide additional insight into the topic area.

Project	Theme	Subtheme	Other Representative Quotes
Beaver Harvesting Activities	Knowledge		*“I think this was the first time I saw this, this wintertime when we were doing the trapping for the beaver and these young guys came out to learn to catch the beaver. I think its like maybe their parents don’t have the experience. Or some of them, I think most people, don’t have the equipment to take their family out. But that can be also from lack of education or lack of experience and at the same time I think they’re not too confident in themselves to take their family out cause they don’t have that experience”* (Participant 3, Elder) *“Yeah man, this was fun man, making all these holes. Well, I didn’t make them, like I had to watch them do it. But it was fun taking the ice out of it. But making these traps was pretty cool man”* (Participant 8, Youth)
		Sharing Knowledge	*“I felt good about learning different things that I didn’t know that I should know maybe for one day…Yeah long as this program keep working, I guess”* (Participant 16, Youth) *“These are the things an Elder has to explain to help…the youth…Because an Elder has to teach, has to educate the young generations today, to respect”* (Participant 18, Elder) *“And that’s how I teach in school, I teach them by showing them how to do it and sometimes, some teachers will say do you have a blueprint? It’s in here (points to head) …like to learn they are hands on, eh…So I always have lots of kids in my classrooms, that tells me I’m doing something right”* (Participant 3, Elder)
		Familial and Social Supports	*“I like to feel needed”* (Participant 8, Youth) *“I didn’t really have family…my uncles teach me here and there, but I’m always hanging out with my friends. My uncles take me out hunting and stuff”* (Participant 28, Youth) *“I didn’t really do much trapping with my uncle, just go check his traps mostly”* (Participant 28, Youth)
	Identity	Cultural Connection	*“Maybe through culture… I could feel myself being out there… there’s part of me being out there…I guess that’s just how our people use to be like long time ago”* (Participant 16, Youth)
		Traditional Activities	*“Use to go out almost every year… every spring, and just recently I started going moose hunting* (Participant 10, Youth) *“For me it’s like spending lots of time out on the land… depends on the season I still live my life based on our cycles. There are six cycles in our in our traditional culture. So, I live by that by that calendar…In the springtime it’ll be like spring hunting, and then I sort of sometimes just before breakup it’s always, like there’s the hard crust and you can travel long distance, as opposed supposed to in the wintertime it’s soft snow, right? So, in spring there’s this crust on top of the snow that you can, it’s like a pavement (laughs)”* (Participant 3, Elder)
	Healing	Physical and Emotional Healing	*“She (daughter) loves being outside, she feels free to run around, she likes the air, she likes the wide-open space, she always has that big smile on her face and that’s the thing about her like, she likes to be out there”* (Participant 21, Youth) *“I think more activities…that’s what we need and, and more training …to keep everyone busy throughout the summer”* (Participant 21, Youth) *“When he’s stuck at home, he goes in the bush, two three days and he’s better…Clean air, being active, yep lot of walking, but at home you don’t really walk, you just sit, and there’s no air. I mean not as clean as out here”* (Participant 36, Elder) (English Translation from Cree) *“If it’s measured can be from just the difference from being out there like say if I was out there, and I had to get my blood pressure checked…I know it would be low, but then coming back cause it’s so busy here and you know you’re on the move. It’s not like you’re in the big city, it’s crazy in the big city, when everything’s moving, you’re always in a rush eh”* (Participant 33, Expert) *“I used to go fishing around there…I use to go by canoe or the long trails, I’d get there by walking”* (Participant 28, Youth). *“I walk on the dyke sometimes but after breakup, I go duck hunting, birds are flying around for a while”* (Participant 28, Youth). *“Doing all kinds of stuff, hunting, you know carvings…I do that too…moose carvings…You know Martens? I sell those fur eh…We send them out in (omitted)”* (Participant 12, Expert)
	Land	Place	*“I just felt the nature more in the bush, than coming out in the community, just felt different. Feeling the nature, feels different”* (Participant 16, Youth) *“It was really nice being out there… gets your mind off a lot of things too. Being out, out in the bush”* (Participant 16, Youth)
Beaver Dam Removal	Knowledge	Sharing Knowledge	*“There was an Elder that use to teach young people, and one day… well he was telling me a story he said, you know we have this last year we had these young people come here to trap, there were lots of them, and he said, but then he told me, but you know what, even if you have 200 students, and you taught them, but you only manage to teach one, you’ve done your job, because that one person will continue that eh? He’ll do the same thing. So, he said it doesn’t matter how many you have, as long as you can reach one then you’ve done something, you’ve passed what you need to do”* (Participant 3, Elder)
		Familial and Social Supports	*“You should never tell a child that their wrong because they’re just expressing their belief the way they’ve been brought up to believe it eh? Their worldview is like, they’re growing up into it they’re just starting to understand, so they don’t know what’s wrong or right but their expressing it. so therefore, you don’t have a right to tell them no that’s not right, you don’t do that… that’s one of the difficulties I have… When I can’t tell someone that’s a mistake, I can’t it’s hard for me to do that. So, I don’t…When I tell my students, you write something, tell me how you understand it, and I’m all ears for it. but I’m not going to mark you wrong. Because there’s no right or wrong. It’s just you explain how you see it, the way you understand it. Because I’m not going to understand how, if we look at the same thing, you going to say something different about it, I’m going to say something different based on how I see it”* (Participant 3, Elder)
		Healing	*“Just feels good…Just like to go out hunting”* (Participant 11, Youth)
	Land	Place	*“We also believe that everything is spiritual, eh? That everything is alive. So that’s why when you when you pick something from a tree you should always thank the tree or you thank the animal for giving up its life so you can you can live… for us everything is alive, doesn’t matter what it is, it can be a rock can be a tree everything that we see is alive, even the smallest uh plant, everything it has a special reason to be here so, sometimes we take a medicine that help us, before I take the medicine you should always talk to the plant as if it were a person you give thanks, for what it’s going to do to you… its purpose”* (Participant 3, Youth)
